# The effect of arterial spin labeling MR angiography (ASL-MRA) in visualizing the branches of external carotid artery

**DOI:** 10.1038/s41598-024-55018-4

**Published:** 2024-02-24

**Authors:** Akira Yogi, Junji Ito, Kazuki Ishikawa, Joichi Heianna, Satoshi Sakugawa, Narihisa Aguni, Makoto Obara, Hiroyuki Maeda, Akihiro Nishie

**Affiliations:** 1 Department of Radiology, University of the Ryukyus Hospital, 207 Uehara, Nishihara-Cho, , Nakagami-Gun, Okinawa 903-0125 Japan; 2 Department of Radiology, Graduate School of Medical Science, University of the Ryukyus, 207 Uehara, Nishihara-Cho, Nakagami-Gun, Okinawa 903-0215 Japan; 3 Department of Radiology, Nanbu Tokushukai Hospital, 171-1 Hokama Yaese-Cho, Shimajiri-Gun, Okinawa 901-0493 Japan; 4 Philips Japan Healthcare, 13-37, Kohnan 2-Chome, Minato-Ku, Tokyo, Japan; 5 Department of Otorhinolaryngology, Head and Neck Surgery, Graduate School of Medical Science, University of the Ryukyus, 207 Uehara, Nishihara-Cho, Nakagami-Gun, Okinawa 903-0215 Japan

**Keywords:** Medical research, Head and neck cancer

## Abstract

This study aimed to assess the performance of arterial-spin labeling MRA (ASL-MRA) for visualizing the external carotid artery (ECA) branches in comparison with time-of-flight MRA (TOF-MRA) and CT angiography (CTA). We retrospectively selected 31 consecutive patients, who underwent both MRAs and CTA, prior to the intra-arterial chemoradiotherapy (IACRT) for head and neck cancer. Four patients underwent IACRT bilaterally, so we analyzed 35 ECAs. Pseudo-continuous, three-dimensional ASL using a turbo field echo sequence was acquired. For the TOF-MRA and CTA, clinically used parameters were applied. Two observers evaluated each ECA branch with reference to the angiogram at the IACRT, using five-point scale, in consensus. Friedman test for multiple comparisons was applied. ASL-MRA and CTA better visualized the superior thyroid, lingual, facial, submental, transverse facial, and internal maxillary arteries (IMAs) better than TOF-MRA (*p* < 0.05). In addition, CTA was superior to ASL-MRA in visualizing only submental artery among these arteries (*p* = 0.0005). Alternatively, the ASL-MRA was superior for visualizing the middle meningeal artery (MMA) and IMA, compared to the CTA (*p* = 0.0001 and 0.0007, respectively). ASL-MRA was superior to the TOF-MRA and similar to the CTA in visualizing most of ECA branches. Furthermore, ASL-MRA can better visualize the periphery of MMA and IMA than CTA.

## Introduction

Head and neck cancers comprise the seventh most common cancer globally and account for over 800,000 new worldwide cases annually^1,2^. Treatment consists of various combinations of surgery, radiation, and chemotherapy according to the TNM Classifications of Malignant Tumors and primary site^3^. Intra-arterial chemotherapy combined with radiotherapy (IACRT) provides favorable local control and survival rates in patients with head and neck cancer as surgical resection is followed by chemoradiotherapy^4^. Understanding the anatomy of the external carotid artery (ECA) is crucial for angiographers before a procedure.

CT angiography (CTA) is an useful tool to evaluate the ECA system^5,6^. Although CTA can provide detailed information of vascular structures with high spatial resolution, some factors inhibit or reduce its utility. For example, in patients with severe renal dysfunction, contrast-enhanced materials may not be administered. Additionally, dental implants often cause metallic artifacts and interfere with the correct visualization of the head and neck.

Time-of-flight MR angiography (TOF-MRA) is one of the most widely used noninvasive MR sequences for observing cerebral arteries. In the head and neck, the common and internal carotid, and vertebral arteries are also well visualized. In patients with head and neck cancer with dental implants or severe renal dysfunction, TOF-MRA could be a better option than CTA^7,8^. However, it is not optimal for showing slow flowing vasculature, or blood flow moving inferiorly and/or horizontally, which are often features of many ECA branches. Therefore, the derived MRA image may not be able to visualize the ECA system as sufficiently as CTA^9^.

Recently, MRA using the arterial spin labeling (ASL) method (ASL-MRA) has been used clinically to visualize cerebral arteries. ASL-MRA can show small intracranial arteries that flow slowly and inferiorly^10^. Thus, ASL-MRA can visualize the distal arteries and collaterals of Moyamoya disease and dural arteriovenous fistulas better than TOF-MRA^11–13^. However, to the best of our knowledge, the performance of ASL-MRA in visualizing ECA branches has not yet been investigated. In addition, it is unknown whether ASL-MRA can visualize ECA branches as well as CTA. We hypothesized that ASL-MRA could also visualize ECA branches better than TOF-MRA. The purpose of this study was to assess the performance of ASL-MRA for visualizing the ECA branches in comparison with TOF-MRA and CTA.

## Materials and methods

### Patients

The University of the Ryukyus Institutional Review Board of approved this study. Due to the retrospective nature of the study, University of the Ryukyus Institutional Review Board waived the need for obtaining informed consent. All methods were performed in accordance with the relevant guidelines and regulations.

Data from patients who underwent IACRT for head and neck cancers at our institute between April 2020 and November 2021 were retrospectively analyzed. Patients who did not undergo two MRAs prior to IACRT, refused imaging, or had poor image quality were excluded.

### MR acquisition

All patients underwent MR examination using a 3 T scanner (Ingenia Elition, Philips Healthcare. Best, The Netherlands). Pseudo-continuous ASL-based three-dimensional (3D) MRA using 3D T1 turbo field echo acquisition was used for ASL-MRA. Voxel size was 1.1 × 1.1 × 1.1 mm^3^. Echo time (TE) and repetition time (TR) were 5.1 ms (ms) and 1.67 ms, respectively. The flip angle was 11°, and the field of view (FOV) was 200 × 200 × 110 mm. The compressed sensing (C-SENCE) factor was seven. The Labeling plane was set parallel to the acquisition orientation and located 30 mm inferior to the carotid artery bifurcation. The labeling duration was set to 2,000 ms, exceeding the in vivo T1 value of blood (approximately 1,600 ms)^12,14^ but remaining within the recommended range for cerebral ASL perfusion imaging^15^. This choice aimed to optimize the capture of labeled arterial signals from the periphery of ECA branches. The post labeling delay (PLD) was set to a minimum value of 50 ms to minimize the risk of missing the labeled arterial signals from the superior thyroid arteries, which often originate around the bifurcation of the common carotid arteries. The total scan time was five minutes and 50 s. A clinically used scan parameter was applied according to TOF-MRA. TR and TE were 23 ms and 3.5 ms, respectively. Flip angle was 18. FOV was 220 × 220 × 110 mm. Voxel size was 0.55 × 0.8 × 1.1 mm. The C-SENCE factor was four. The total scan time was four minutes and 41 s. Note that the voxel size was slightly larger on ASL-MRA.

### CT acquisition

Sublingual nitroglycerin administration was administered to all patients prior to CT examination to enhance the periphery of the ECA system^16^. This was not performed on either MRAs. CTA was performed using a 160 multidetector row system (Aquilion Precision; Canon Medical Systems, Tochigi, Japan). The detector matrix was 1792 channels × 160 rows, and each detector element was 0.25 × 0.25 mm at the isocenter. The beam collimation was 0.25 mm × 160 mm at the isocenter. The small focal spot of the X-ray tube was at 100 kV and 310 mA. The image acquisition in super-high resolution mode was as follows: slice thickness, 0.5 × 512 row helical scan; tube voltage, 100 kVp; tube current, set by the automatic tube current setting; rotation time 0.5 s, beam pitch 0.806, and 512 × 512 reconstructed matrix. The volume CT dose indexes (CTDI_vols_) and dose length product (DLP) were 29.5 ± 8.7 mGy and 294.3 ± 77.3 mGy, respectively.

Contrast medium (Iopamiron®; Bayer Yakuhin, Osaka, Japan) was injected at an equivalent dose of 370 mg iodine per milliliter (mgI/mL) at a rate of 25.0 mgI/kg/s for 10 s, followed by a 30 mL saline flush injected at the same rate using a dual-head injector (Nemoto Kyorindo, Tokyo, Japan). Established venous access was performed in the right basilic vein according to the scanning protocol. CTA scanning was initiated using a manual bolus tracking technique applied to the terminal of the common carotid artery.

### Angiography

Digital subtraction angiography (DSA) was performed using a standardized clinical protocol on a biplanar system immediately prior to intra-arterial chemotherapy. Frontal and lateral views were obtained after injection of a bolus of iodinated contrast-enhanced material manually in the ECA and ECA branches, as needed.

### Visual evaluation

**Figure 1:**
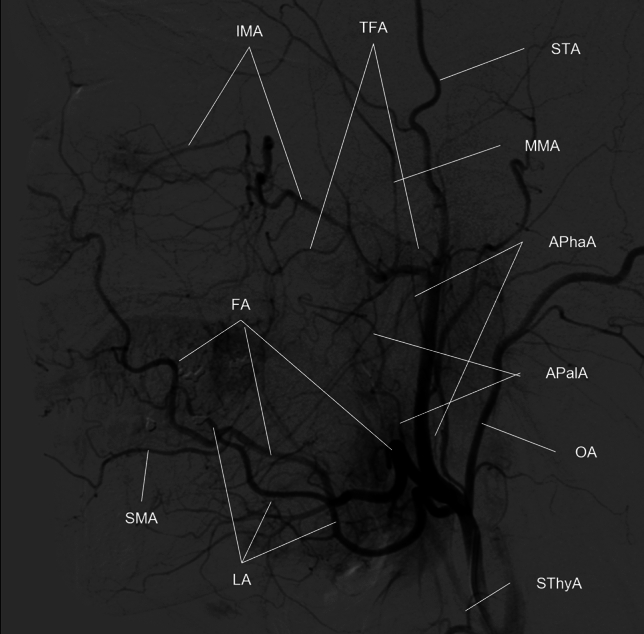
Evaluated branches of the external carotid artery. *APalA* ascending palatine artery, *APhaA* ascending pharyngeal artery, *FA* facial artery, *IMA* internal maxillary artery, *LA* lingual artery, *MMA* middle meningeal artery, *OA* occipital artery, *SMA* submental artery, *STA* superior temporal artery, *SThyA* superior thyroid artery, *TFA* transverse facial artery.

**Figure 2:**
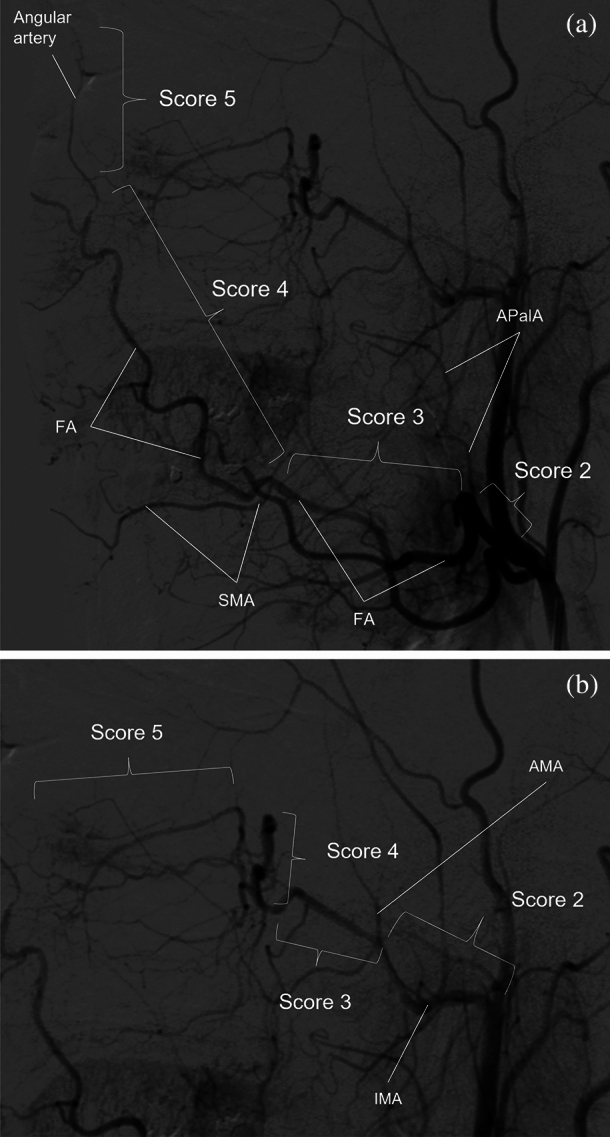
Grading system for the evaluation of the facial and internal maxillary arteries. (**a**) Grading system for the facial arteries. Score 1: “Not visualized” (not shown in the figure), Score 2: “Visualized until the branching point of APalA”, score 3: “Visualized until the branching point of SMA”, score 4: “Visualized over the branching point of SMA”, and score 5: “Angular artery, a terminal branch of FA is visualized”. (**b**) Grading system for the internal maxillary arteries. Score 1: “Not visualized”, score 2: “1st segment is visualized”, score 3: “2nd segment is visualized”, score 4: “3rd segment is visualized”, and score 5: “Any of branching arteries at 3rd segment is visualized”. *AA* angular artery, *AMA* accessory meningeal artery, *APalA* ascending palatine artery, *FA* facial artery, *IMA* internal maxillary artery, *SMA* submental artery.

**Figure 3:**
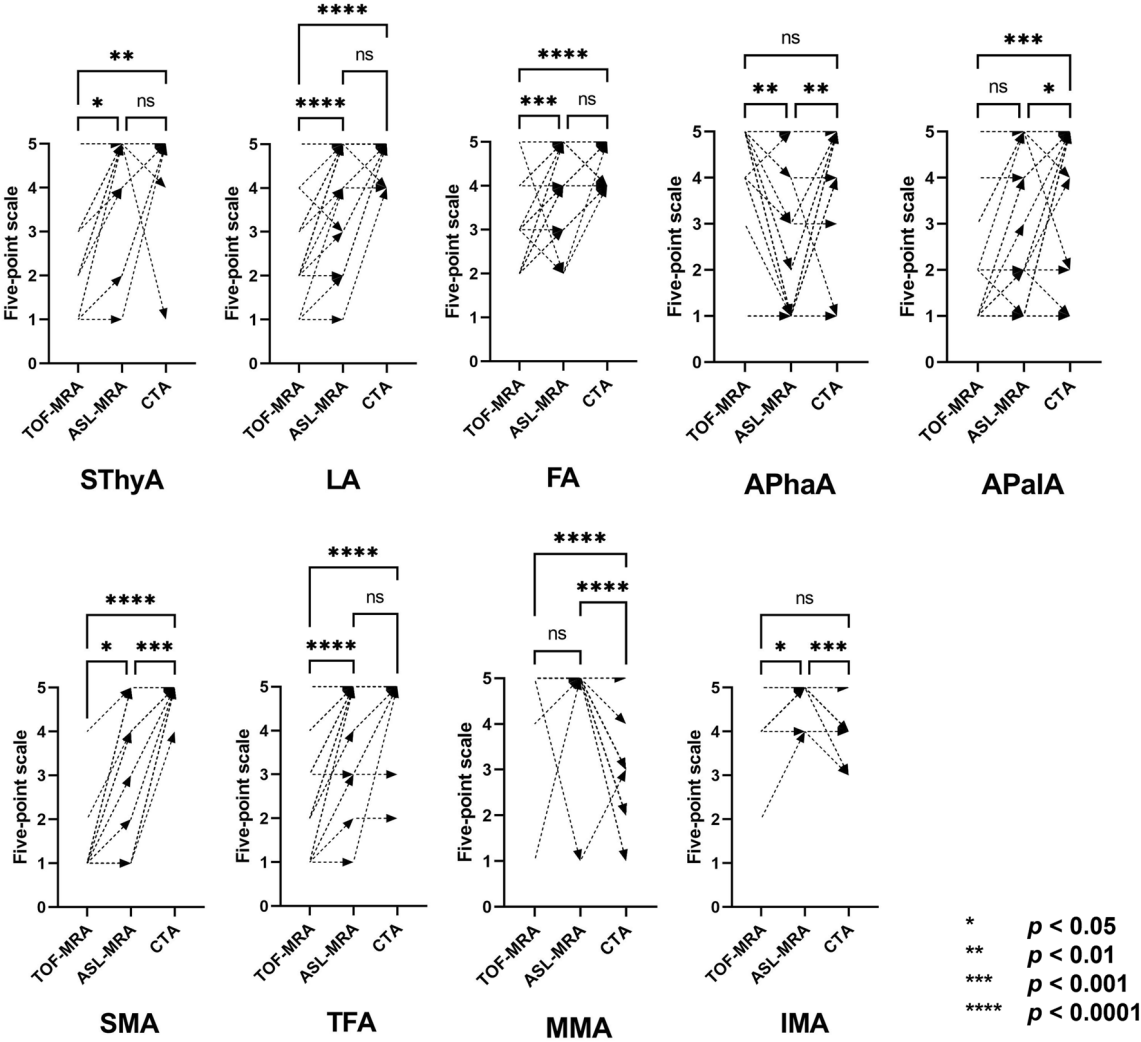
Comparison of visualization scores among TOF-MRA, ASL-MRA and CTA. *Apala* ascending palatine artery, *APhaA* ascending pharyngeal artery, *FA* facial artery; *IMA* internal maxillary artery, *LA* lingual artery, *MMA* middle meningeal artery, *OA* occipital artery, *SMA* submental artery, *STA* superior temporal artery, *SThyA* superior thyroid artery; transverse facial artery.

Maximum intensity projection (MIP) images were generated for both MRAs and CTAs using all acquired slices. The contralateral side of the head and neck was trimmed on the MIP images to avoid overlapping of the bilateral ECAs during evaluation. Bony structures were automatically excluded from the CTA MIP image using a commercially available workstation (Synapse Vincent; FUJIFILM Medical Co., Ltd., Lexigton, MA, USA). To avoid bias, we did not edit the MIP images. The superior thyroid artery (SThyA), lingual artery (LA), facial artery (FA), ascending palatine artery (APalA), submental artery (SMA), occipital artery (OA), ascending pharyngeal artery (APhaA), internal maxillary artery (IMA), middle meningeal artery (MMA), transverse facial artery (TFA), and superficial temporal artery (STA) were evaluated by both MRAs and CTA using DSA images as a reference standard (Fig. 1). We built a five-point scale for each artery, except for FA and IMA, as follows: score 1, no visualization; score 2, poor visualization, less than 25% of the artery was visualized; score 3, moderate visualization, 25–50% of the artery was visualized; score 4, good visualization, 50–75% of the artery was visualized; and score 5, excellent visualization, where 75–100% of the artery was visualized. We defined the following grading system for the evaluation of FA using FA branching arteries, which could be used as a landmark in angiography (Fig. 2). For FA, we defined score 1 as “not visualized”; score 2 as “visualized until the branching point of APalA”; score 3 as “visualized until the branching point of SMA”; score 4 as “visualized over the branching point of SMA”; and score 5 as “angular artery, a terminal branch of FA is visualized”. We built the grading system of the IMA based on the following anatomical segments: mandibular (1st.), pterygoid (2nd.), and pterygopalatine (3rd.) segments, respectively (Fig. 3)^17^. The mandibular segment runs vertically at first, turns horizontally, and then makes an acute turn anteriorly, which is the transition to the pterygoid segment. The MMA, inferior alveolar artery, and accessory meningeal artery branched in this segment. The pterygoid segment runs across the lateral pterygoid muscle and turns anteromedially before entering the pterygopalatine fossa. The middle deep temporal artery was located in this segment. The pterygopalatine segment turned transversely at the entrance of the pterygopalatine fossa and ran to the superior fossa. It branches into the posterior superior alveolar artery, infraorbital artery, descending palatine artery, sphenopalatine artery, pharyngeal artery, foramen rotundum artery, and Vidian artery. Based on this anatomical segmentation, we defined the visualizing score one, two, three, four and five as “not visualized”, “1st segment is visualized”, “2nd segment is visualized”, “3rd segment is visualized”, and “any of the branching arteries at the 3rd segment are visualized”, respectively.

Two board-certified radiologists blindly evaluated the visualization of each ECA branch on the MIP images in a random order, in consensus. The window settings were freely changed by the observers during the interpretation. Discrepancies were resolved through open discussion.

### Statistics

The evaluated MIP images were classified into the ASL, TOF, or CTA groups. Friedman test for multiple comparisons was applied to compare the scores of each branch among the three groups by statistical software (GraphPad Prism 22, GraphPad Software, California, U.S.A.). Statistical significance was set at P < 0.05.

## Results

Between April 2020 and November 2021, 35 patients underwent IACRT for head and neck cancers at our institute. Two patients did not undergo both MRAs prior to the first IACRT, one patient refused MR examination because of claustrophobia, and the other patient was not enrolled due to poor image quality. The remaining 31 consecutive patients who underwent neck TOF-MRA, ASL-MRA, and CT angiography before IACRT were retrospectively selected for analysis. The mean interval periods between MRAs and CTA, MRAs and DSA, and CTA and DSA are 12.5 ± 8.6 days, 1.1 ± 5.0 days, and 13.6 ± 6.9 days. We did not confirm any significant interval change of the cancers during these periods. Four patients underwent bilateral IACRT; therefore, we evaluated 35 ECAs. Demographic data are summarized in Table 1. Visualizing score was summarized in Table 2 and Fig. 3. One patient with right maxillary cancer had the ipsilateral ophthalmic artery arise from the MMA. Intra-arterial chemotherapy was administered distally to the origin of the MMA.

**Table 1:** Patients’ demographic data.

Descriptions	Numbers
Number of cases^†^	31
Gender	Male: 25, female: 6
Age at surgery, years	43—90 (median 65)
Interval between MRAs and CTA, days	12.5 ± 8.6
Interval between MRAs and IACRT, days	2.0 ± 1.3
Diagnosis	
Oropharyngeal cancer	12
Maxillary cancer ^†^	10
Glottic cancer	2
External auditory canal cancer	2
Nasopharyngeal cancer	1
Hard palate cancer	1
Soft palate cancer	1
Gingival cancer	1
Nasal cancer	1

*CTA* computed tomography angiography, *IACRT* intra-arterial chemotherapy combined with radiotherapy, *MRA* magnetic resonance angiography.

^†^Ten maxillary cancers consisted of nine squamous cell carcinomas and one adenoid cystic carcinoma.

**Table 2:** Visualization scores of ECA branches on both MRAs and CTA.

	TOF-MRA^†^	ASL-MRA^†^	CTA^†^	ASL-MRA vs. TOF-MRA^‡^	TOF-MRA vs CTA^‡^	ASL-MRA vs CTA^‡^
SThyA	2.9 ± 1.6	4.4 ± 1.2	4.8 ± 0.9	0.02*	0.006*	> 0.99
LA	2.4 ± 1.0	4.3 ± 1.2	4.9 ± 0.3	< 0.0001*	< 0.0001*	0.66
FA	2.9 ± 0.9	4.1 ± 0.2	4.5 ± 0.5	0.0002*	< 0.0001*	0.76
APalA	1.9 ± 1.5	2.6 ± 1.7	4.1 ± 1.5	0.33	0.0002*	< 0.05*
SMA	1.1 ± 0.6	2.7 ± 1.7	4.7 ± 0.2	0.03*	< 0.0001*	0.0005*
OA	5.0 ± 0.0	5.0 ± 0.0	5.0 ± 0.0	NA^#^	NA^#^	NA^#^
APhaA	4.1 ± 1.5	2.2 ± 1.6	4.3 ± 1.3	0.002*	> 0.99	0.0016*
TFA	2.7 ± 1.3	4.5 ± 0.2	4.8 ± 0.6	< 0.0001*	< 0.0001*	> 0.99
IMA	4.4 ± 0.7	4.9 ± 0.3	4.2 ± 0.6	0.02*	0.95	0.0007*
MMA	4.8 ± 0.7	4.9 ± 0.7	2.6 ± 0.9	> 0.99	< 0.0001*	0.0001*
STA	5.0 ± 0.0	5.0 ± 0.0	5.0 ± 0.0	NA^#^	NA^#^	NA^#^

*APalA* ascending palatine artery, *APhaA* ascending pharyngeal artery, *ASL* arterial spin labeling, *CTA* computed tomography angiography, *ECA* external carotid artery, *FA* facial artery, *IMA* internal maxillary artery, *LA* lingual artery, *MMA* middle meningeal artery, *MRA* magnetic resonance angiography, *OA* occipital artery, *SMA* submental artery, *STA* superior temporal artery, *SThyA* superior thyroid artery, *TFA* transverse facial artery, *TOF* time-of-flight.

^†^Visualization scores are summarized as means and standard deviations.

^‡^*p* values calculated for the comparisons are shown for each cell.

^#^Comparisons were not performed because all OA and STA were rated with a score of 5 on both MRAs and CTA.

*p < 0.05.

### Comparison between TOF-MRA and ASL-MRA

**Figure 4:**
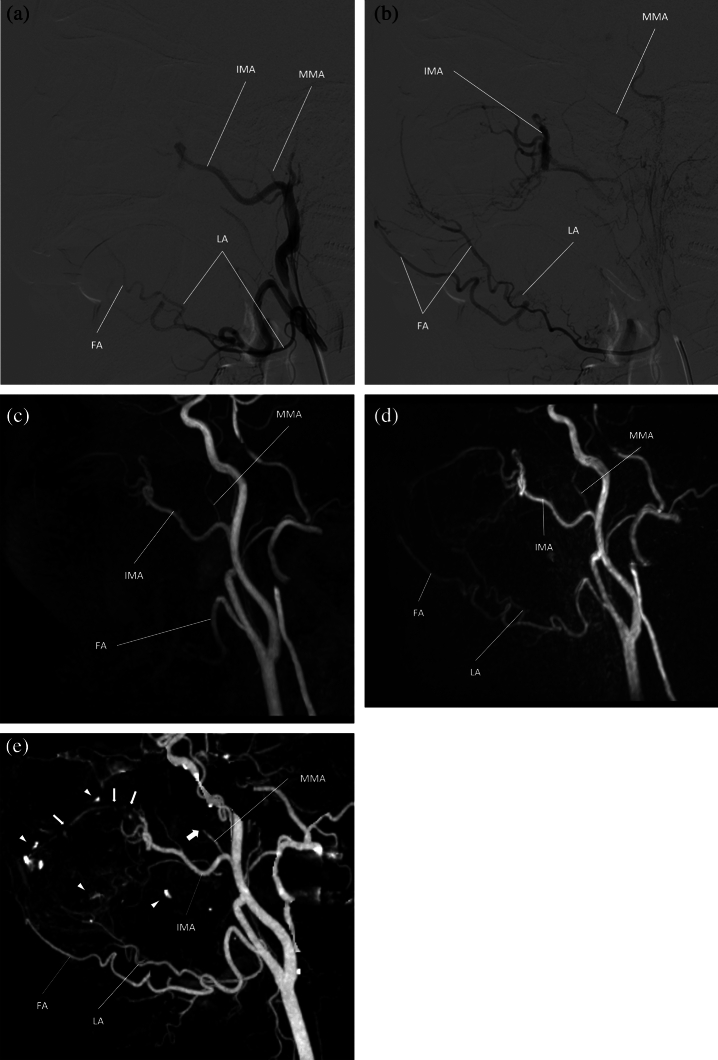
A 67-year-old male with left maxillary sinus cancer. (**a**, **b**) Two consecutive phases of the angiogram at the left external carotid artery. (**c**) On TOF-MRA, the lingual artery (LA) is not visualized (score 1), and the facial artery (FA) is visualized over the branching point of the ascending palatine artery, but the branching point of the submental artery (SMA) is not visualized (score 3). Branches of the 3rd segment of the internal maxillary artery (IMA) is partially demonstrated (score 5). The middle meningeal artery (MMA) is well visualized. (**d**) On ASL-MRA, the LA is demonstrated almost completely (score 5), and the FA is demonstrated over the branching point of the SMA (score 4). More branches of the 3rd segment are visualized (score 5), and the MMA is well visualized (score 5). (**e**) CTA demonstrated the LA and FA clearer than both MRAs (score 5 and 4, respectively). Note that the extent of visualized FA is the same as that on ASL-MRA. CTA demonstrates the branches of the 3rd segment as well as ASL-MRA (score 5); however, the visualization of the 3rd segment is inferior to ASL-MRA, because there are some disruptions (thin arrows) and some spots of contrast enhancement (arrow heads). For the MMA, only the origin is visualized on CTA (thick arrow).

**Figure 5:**
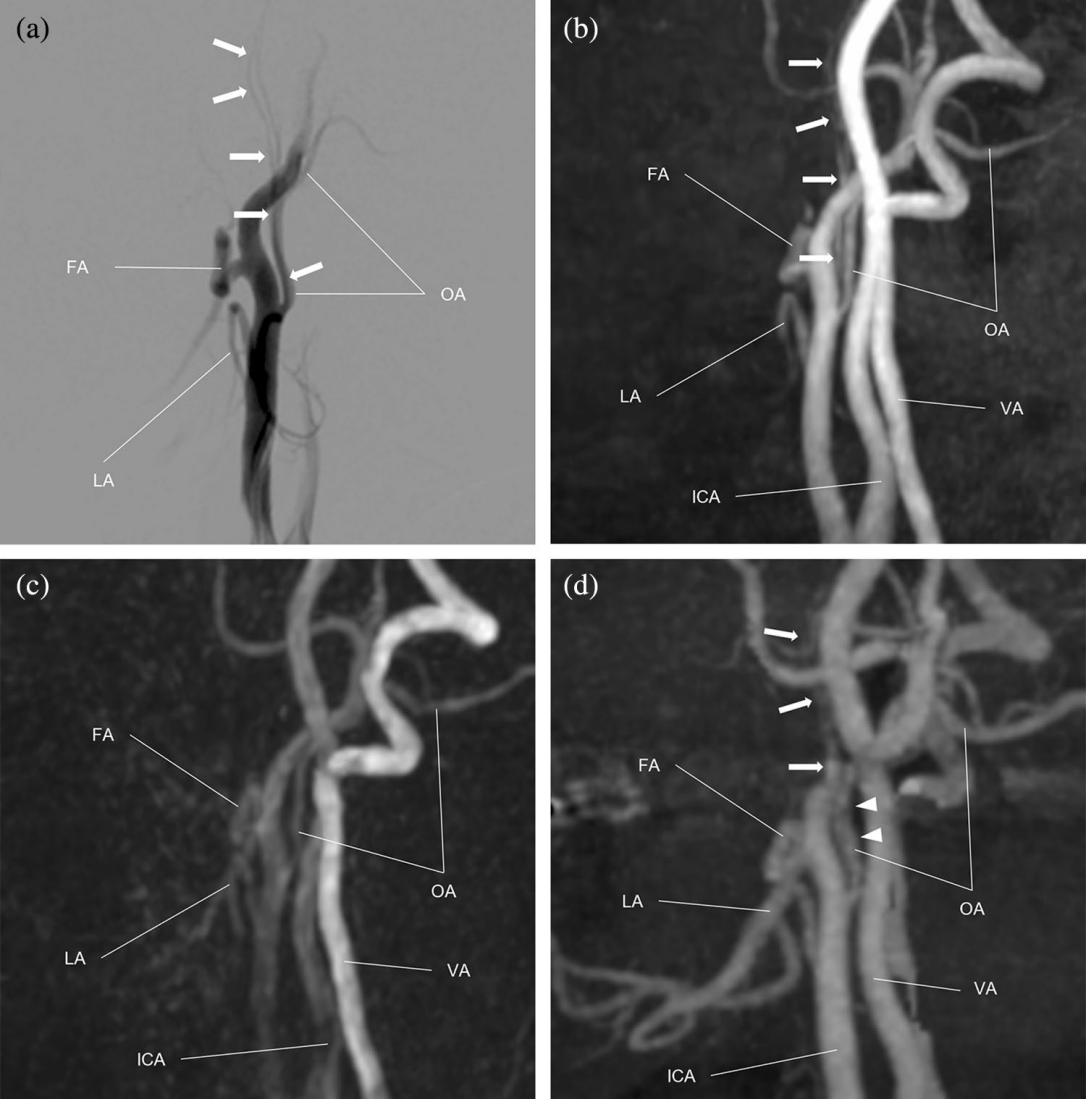
A 65-year-old male with left oropharyngeal cancer. (**a**) An early phase of the angiogram at the left external carotid artery. The ascending pharyngeal artery (APhaA) arises at the origin of the occipital artery and almost directly runs superiorly (arrows) (**b**) TOF-MRA demonstrates the APhaA entirely (arrows) and is rated as a score 5. (**c**) ASL-MRA does not demonstrate the APhaA at all (score 1). (**d**) CTA also demonstrates the APhaA as well as TOF-MRA (score 5). There are, however, it is hard to differentiate the proximal of APhaA from OA (arrow heads), so that the visualization quality for APhaA is slightly less than that of TOF-MRA.

ASL-MRA visualized SThyA, LA, FA, SMA, TFA and IMA better than TOF-MRA (*p* = 0.02, < 0.0001, 0.0002, 0.03, < 0.0001 and 0.02, respectively) (Fig. 4). There were no significant differences in the visualization of the APalA and MMA (*p* > 0.33). ASL-MRA was inferior to TOF-MRA in visualizing only the APhaA (*p* = 0.002) (Fig. 5).

### Comparisons between MRAs and CTA

TOF-MRA was inferior to CTA for visualization of the SThyA, LA, FA, APalA, SMA, and TFA (*p* = 0.006, < 0.0001, < 0.0001, 0.0002, < 0.0001, and < 0.0001, respectively) (Fig. 4). There was no significant difference in the visualization of APhaA and IMA between TOF-MRA and CTA (Fig. 5).

ASL-MRA visualized the MMA and IMA better than CTA (*p* = 0.0001 and 0.0007, respectively) (Fig. 4). The proximal part of the MMA was well visualized on CTA; however, the MMA in the spinous foramen, which is a narrow canal surrounded by a bony structure, was excluded, as was the cranium at the bone subtraction process in the workstation (Fig. 4e). Scores of IMA rated on CTA were higher than three but were equal to or lower than those rated on ASL-MRA. ASL-MRA was inferior to CTA in visualizing the APhaA (Fig. 5), APalA, and SMA (*p* = 0.002, < 0.05, and 0.0005, respectively). There was no significant difference in the visualization of SThyA, LA, FA, and TFA between ASL-MRA and CTA. All OAs and STAs were scored as five on both MRAs and CTA.

## Discussion

In this study, ASL-MRA was superior to TOF-MRA for visualizing many ECA branches, including SThyA, LA, FA, SMA, TFA, and IMA. ASL is a non-invasive method for evaluating cerebral blood flow by labeling the magnetization of arterial blood using radiofrequency pulses, which have labeled blood as an endogenous tracer^13^. The ASL-MRA technique does not depend on the inflow effect; therefore, it is useful for visualizing distal arteries and collaterals in Moyamoya disease and dural arteriovenous fistulas, which TOF-MRA cannot precisely visualize^11–13^. As many ECA branches also run in various directions with meandering, it is not surprising that ASL-MRA performs better than TOF-MRA.

In contrast, TOF-MRA visualized APhaA better than ASL-MRA. APhaA is small, with a mean diameter of 1.2 mm ^18^, which may be too small for ASL-MRA visualization with an isotropic voxel size of 1.1 mm. This artery is also small for TOF-MRA; however, the anatomical characteristics of the APhaA, which flows in a superior direction without any meandering, might allow TOF-MRA to obtain a sufficient in-flow effect for visualization.

Furthermore, for many ECA branches, ASL-MRA was similar to CTA for visualization. In addition, ASL-MRA was superior to CTA for visualizing the periphery of the IMA and MMA. Notably, lingual administration of nitroglycerin was not performed on ASL-MRA but was for CTA. These results demonstrate the high performance of ASL-MRA in visualizing the ECA branches.

The MMA is typically described as the largest ascending branch of the IMA and supplies more than two-thirds of the cranial dura^19^. The mean diameter of the extracranial part of the MMA ranges from 2.6 to 2.9 mm ^20^ and runs in the superior direction without strong meandering; because of this, the MMA was well visualized on both MRAs. In contrast, only the proximal part of the MMA before entering the spinous foramen was visualized in all cases on the MIP image of CTA. Because the spinous foramen is narrow and a tiny hole surrounded by the cranial bone, the bone subtraction function in the workstation could not differentiate the MMA in the spinous foramen from the cranial bone, resulting in the exclusion of the artery with bony structure. The MMA is one of the most important arteries according to the anastomosis between the internal and external carotid arteries^21,22^. In particular, the ophthalmic artery sometimes originates from the MMA^23^; therefore, it is important to know whether the ophthalmic artery arises from the MMA to avoid administering anticancer agents into the ophthalmic artery. In this study, one patient demonstrated the right ophthalmic artery arising from the ipsilateral MMA, so that we could perform the intra-arterial chemoradiotherapy the distal part from the origin of MMA. It may be possible to manually re-generate the excluded part of MMAs in some cases, using the “region growing” function of the workstation^24^; however, this is operator dependent. Because both TOF-MRA and ASL-MRA may be able to visualize these anastomoses easily and consistently, they might be more appropriate for screening variations prior to the procedure.

ASL-MRA was also superior to CTA in visualizing the IMA. The IMA scores rated on CTA were higher than three and equal to or lower than those rated on ASL-MRA. Therefore, ASL-MRA was superior in visualizing the 3rd segment of the IMA, which starts at the pterygopalatine fossa, runs transversely to the superior fossa, and branches into some arteries. At the 3rd segment, the IMA and branching arteries run close to the paranasal bones, similar to the MMA. Therefore, it is also assumed that the 3rd segment of the IMA might be excluded from the MIP image with bones during the bone subtraction process. It is important to evaluate the 3rd segment of the IMA because the Vidian artery, one of the branching arteries there, could be an anastomosis between the IMA and internal carotid artery^17^. Considering the visualizing performance of the MMA and 3rd segment of the IMA, ASL-MRA might be the best pre-procedural imaging examination in patients who will undergo IACRT from the IMA, including maxillary cancer.

In contrast, ASL-MRA was inferior to CTA in visualizing the APhaA, APalA, and SMA. The APalA and APhaA are small arteries, with mean diameters ranging from 0.4 to 0.7 mm ^25^, and ASL-MRA may not be able to consistently visualize them. Surprisingly, the SMA, which is prominent and does not run superiorly, was not well visualized on ASL-MRA. It is supposed that motion artifacts in the oral cavity due to swallowing during the examination may interfere with visualization. Because metallic artifacts such as dental implants often interfere with visualization of the SMA, a new technique that is not affected by metallic artifacts or motion artifacts is necessary.

Furthermore, TOF-MRA was inferior to CTA in visualizing many ECA branches, including the SThyA, LA, FA, APalA, SMA, and TFA. This result is similar to that reported by Cappabianca et al.^9^ and supports previous knowledge.

Finally, the OA and STA were completely visualized on both MRAs and CTA images. Both arteries are typically prominent and run in the direction superiorly without meandering. In addition, there is nothing to interfere with the visualization, such as metallic artifacts or motion artifacts. Therefore, contrast-enhanced materials are not required to evaluate these two arteries.

This study has several limitations. Contrast enhanced TOF-MRA with gadolinium injection, which could visualize the periphery of ECA system more, was not performed on our patients, because of the examination time and the slight invasive method. However, contrast enhanced TOF-MRA visualize not only small arteries, but also the venous system, so that it is hard to visualize only ECA branches separately^26^. Therefore, contrast enhanced MRA would not be superior to ASL-MRA, which can visualize only the periphery of ECA system. The window settings were not uniform among both MRAs and CTA for image interpretation. However, it is difficult to standardize the window settings because the imaging modalities and sequences are quite different. Window settings of MIP images are usually changed to determine the best setting for visualization of the whole vascular structure in clinical practice and were regularly changed freely. Image interpretation was not performed separately; therefore, we could not evaluate the reproducibility of scoring using intraclass correlation analysis. This is a retrospective study with a relatively small sample size of 35 ECAs in 31 patients. Consequently, the present results may be susceptible to bias. Given the preliminary nature of this study, further prospective evaluations with larger sample sizes are warranted.

## Conclusions

ASL-MRA was superior to TOF-MRA and similar to CTA in visualizing most of the ECA branches. Furthermore, ASL-MRA can better visualize the periphery of the MMA and IMA than CTA. ASL-MRA may provide the angiographer additional information on vascular anatomy, including anastomosis between the ECA and ICA, in patients with head and neck cancers. ASL-MRA may, therefore, have clinical value in managing patients with head and neck cancer.

## Data Availability

The datasets analyzed during the current study are available from the corresponding author on reasonable request.
